# Ageing in place in urban environments: building collaborative organisations for later life

**DOI:** 10.1007/s10433-025-00875-9

**Published:** 2025-09-16

**Authors:** Tine Buffel, Chris Phillipson

**Affiliations:** University of Manchester, Manchester, UK

**Keywords:** Ageing in place, Collaborative organisations, Urban environments, Older people, Age-friendly cities

## Abstract

Ageing in place, which refers to the idea of supporting older adults to remain living in their homes and communities for as long as they wish, has emerged as a dominant approach in public policy. However, the wider urban context influencing *how* people age in place, together with the pressures on the *places* in which people age, has been the subject of much less discussion. In response, this article reviews different ways of supporting ageing in place, exploring this theme in the context of issues associated with widening urban inequalities, austerity policies, and the privatisation of services and spaces within cities. Building on this analysis, the paper assesses the potential of initiatives which can empower and support groups within the older population, highlighting developments such as the village model, naturally occurring retirement communities, cohousing, and compassionate communities. Reflecting on the strengths and limitations of these initiatives, the paper examines the possibilities for developing new approaches to ageing in place *as a collaborative venture*, drawing on the collective resources of older people themselves, transforming as a result the urban environments in which they are themselves key actors.

## Introduction

‘Ageing in place’ has emerged as a dominant theme in public policy, reflecting the shift towards community-based care, and renewed emphasis on the role of neighbourhoods in contributing to well-being in later life. It has run parallel with attempts to create what the World Health Organization (WHO 2018) has defined as ‘age-friendly cities and communities’, or supportive social and physical environments for people of all ages. However, as this paper argues, the implementation of ageing in place policies coincided with the introduction of austerity measures, the scaling back of the welfare state, and a shift towards the privatisation of services and spaces within cities. In consequence, the pressures on the *places* in which people are ageing have intensified, with many areas experiencing rising inequalities, deteriorating physical infrastructure, and social divisions within communities (Florida 2017; Savage 2021).

Given this context, the paper aims to understand the structural pressures affecting efforts to promote ageing in place, examine the potential of collaborative organisations designed to empower older adults within their communities, and consider new strategies to address the limitations of current initiatives. This paper offers a novel contribution by proposing a conceptualisation of ageing in place as a broad social and community endeavour—a *collaborative venture—*rather than an individual route into old age. It argues for a shift from focussing solely on individual or familial aspects to a broader perspective that examines how older adults develop, sustain, and mobilise community connections. The paper will, first, situate its review within the context of definitions of ageing in place; second, link these to pressures which are operating within the context of urban environments, where the majority of older people live both now and into the future; third, summarise the potential for developing collaborative organisations which might overcome some of the constraints associated with ageing in place, reviewing their strengths and limitations; finally, put forward some key questions for developing a new agenda for future work around ageing in place.

## Defining ‘ageing in place’

The term ‘ageing in place’ has been used and defined in a variety of ways, building on a large body of work in environmental and geographical gerontology aimed at understanding, explaining, and enhancing the relationship between older adults and their physical surroundings (Wahl and Oswald 2010; Oswald et al. 2024; Andrews 2024). At its core, ageing in place refers to the idea of supporting older adults to remain in their own homes and communities as they age, maintaining a sense of independence, continuity, and familiarity in their lives (Cutchin and Rowles 2024). This concept reflects both a personal aspiration for many older adults and a societal goal supported by various policy and community development initiatives, driven in part by policymakers’ recognition of its financial advantages over residential care (Buffel and Phillipson 2024).

Researchers have approached ageing in place from different perspectives, each emphasising distinct aspects of the concept: some focus on maintaining a degree of *independence*, while others emphasise the importance of *community and social connections*. An example of a definition that focuses on independence comes from Horner and Boldy (2008, p. 356) who define ageing in place as a ‘positive approach to meeting the needs of the older person, supporting them to live independently or with some assistance for as long as is possible’. Golant (2015) introduces the idea of ‘ageing in the right place’ as the process of living in an environment, whether familiar or new, that optimally support older adult’s changing needs, autonomy and well-being. The World Health Organization (2020, p. 37) underscores the importance of the community dimension in its definition of ageing in place, describing it as ‘remain[ing] at home in their [older people’s] familiar surroundings and maintain[ing] the relationships that are important to them’.

Policies to support ‘ageing in place’ have been reinforced by work developing age-friendly cities and communities (AFCC). AFCC initiatives focus on creating environments where older adults are actively involved, valued, and provided with infrastructure and services that meet their needs and aspirations (WHO 2007; Buffel et al. 2024). This includes interventions in the physical environment, such as improving the accessibility of housing, transportation, and health and community services, as well as initiatives in the social environment, such as community engagement programmes and social support networks. Additionally, AFCC emphasises the integration of technology to enhance connectivity, safety, and accessibility for older residents, thereby promoting their ability to remain in their homes and communities while ageing (van Hoof et al. 2021; Marston et al. 2023; Golant 2024).

While the concept of ageing in place is widely embraced, it is essential to critically examine its limitations and challenges. One key concern is the tendency of scholars, practitioners, and older adults themselves to view ‘ageing in place’ as the prevailing standard for ‘ageing well’. However, research shows that ageing in place is not a continuous, uniform solution; it varies in its ‘do-ability’ depending on people’s evolving needs across the life course (Hillcoat-Nallétamby and Ogg 2014). Smetcoren (2015) demonstrates that when older adults’ housing no longer meets these changing needs, it can lead to adverse health and well-being outcomes. Additionally, the feasibility of ageing in place is influenced not only by the changing needs of individuals but also by the characteristics of the *places* where they age. Yet, the wider social, political, and economic contexts influencing *how* people age in place, along with the pressures on these *places*, have received much less attention in scholarship on ageing in place (Buffel and Phillipson 2024). Yarker et al. (2023) call for a deeper conceptualisation of *place* in ageing in place research to critically address issues of *inequality* and the *uneven capacity of places* to support ageing in place, especially for groups facing *exclusion* from their environment. Their perspective aligns with views that see people and places as coconstitutive processes, highlighting the dynamic interplay where older adults are influenced by their environments while simultaneously using agency and collective action to shape these spaces (Andrews 2024; Oswald et al. 2024).

Envisioning a future for the study of ageing in place, Rowles and Cutchin (2024) stress the need to more carefully address the diverse manifestations of *exclusion*—institutional, economic, social, racial and ethnic, and cultural barriers—that limit options for older adults to participate in diverse places. One outcome of such exclusion is the situational paradox of wanting but being unable to remain ‘in place’ in a familiar and supportive environment versus being ‘stuck in place’ in an undesirable location because of the absence of, or inability to access, alternatives (Russel 2024). The latter can be the case for example in areas of high deprivation, or in neighbourhoods undergoing gentrification, where older people’s needs are often overlooked, leading to increased risks of isolation and reduced quality of life (Buffel and Phillipson 2019). In this paper, we are especially concerned with how ageing in place interacts with changes in urban environments, arguing that the relationship between the two highlights the need for new forms of organisation that both support and are led by older people themselves. Before addressing this last point, we first consider the impact of urbanisation on ageing in place, highlighting the pressures from increased inequalities within and between urban areas.

## Ageing in place in urban environments

Population ageing and urbanisation are two key forces shaping social and economic life in the twenty-first century. Population ageing is taking place across all countries of the world, albeit at varying levels of intensity. In OECD countries, the population share of those 65 years and over increased from less than 9% in 1960 to more than 17% in 2010 and is expected to reach 27% in 2050. The increase has been particularly rapid among the oldest group, with the share of the population aged 80 and over projected to more than double from 4.6% in 2019 to 9.8% in 2050 (OECD 2021). Of equal significance is the spread and impact of urbanisation, as well as suburbanisation, influencing many aspects of everyday life across all age groups and generations. Over half of the world’s population (55%) resides in cities, projected to rise to about two-thirds by 2050 (United Nations 2019).

The relationship between these two trends—population ageing and urbanisation—is now the subject of increased academic and policy analysis. Indeed, understanding the forces driving urban change is crucial for contextualising ageing in place and refining strategies to maximise its potential (Goldin and Lee-Devlin 2023). Ageing is experienced in a variety of ways in different kinds of urban places and spaces. Yet cities are, for the most part, imagined and structured with a younger, working-age demographic in mind (Handler 2014). Urban environments create many advantages for older people, through providing access to cultural activities, leisure facilities, and different types of medical and social care. At the same time, they may also produce feelings of insecurity, arising from widening economic and social inequalities, and the impact of gentrification and urban regeneration (Buffel and Phillipson 2024).

Wilson (2020, p. 109) reminds us that: ‘Cities are fragile things. Without constant investment, renewal, and civic mindedness their fragmentation is extraordinarily swift’. This seems an accurate comment on the impact of urban changes in the twentieth and twenty-first centuries, stemming from de-industrialisation, the privatisation of physical and social infrastructure, and austerity policies in many European welfare states. Many of these developments came to a head alongside the introduction of attempts to improve the lives of older adults (and other age groups) living in cities (Greenfield and Buffel 2022). Against this, the combined impact of these forces has been to undermine much of what makes living in cities a desirable quality, weakening the ameliorative effects of policy interventions promoting ageing in place. Widening inequality has been a defining characteristic of cities in the opening decades of the twenty-first century. Florida (2017, p. 107) asserts that: ‘The reality is that deep divides and worsening segregation have become a feature, not a bug, of great global cities’. Indeed, despite the economic benefits of the back-to-the-city movement, concentrated urban poverty is on the rise. Inequalities have been reflected in the expansion of gated communities and condominiums for the elite, and the collapse in affordable housing for the majority. However, Savage (2021, p. 234) makes the point that we need to see: ‘Large cities [as] not just products but drivers of inequality’. As a consequence, the goal of supporting people to age well within their homes and neighbourhoods may be challenged by the complex ways in which urban environments change over time, and the social divisions which arise.

Graham (2016, p. 197) examined the new inequalities associated with the construction of luxury tower blocks in cities, noting their use as second homes by wealthy elites and the resulting decline in affordable housing. Commenting on the impact of these trends in New York (but with similarities across many other cities), he argues that the growth of luxury towers symbolises a much broader shift. This shift includes the easing of social obligations and housing regulations, the removal of long-standing rent controls, the eviction of lower-income tenants, the privatisation of public spaces, and the increasing influence of finance and real estate capital on urban planning.

Stein (2019, p. 40) argues that the forces of property present cities with two options: gentrification on the one side or disinvestment on the other. However, the presence of ageing populations or ageing neighbourhoods suggests a viable alternative: communities that reinvent themselves by developing collaborative solutions for ageing in place, in partnership with statutory services, municipal authorities, not-for-profit and voluntary organisations. Kern’s (2021, p. 81) vision is helpful here, asking: ‘how could we create or re-purpose spaces, especially urban spaces, in ways that open up a wide range of possibilities for sustaining and practising the kind of relationships that we think will support us across the life course’.

One response is the idea of building what Neamtan (2005) and Wright (2010, p.193) have termed a ‘social economy’: economic activity that emerges from voluntary associations within civil society and is grounded in the ability to mobilise people for various forms of collective action. The approach developed by Wright and others underlines the need to examine the scope and relevance of alternative forms of social and economic practices and their potential contribution to developing new approaches to support people ageing in place. A range of possibilities exist or are emerging among groups of older people, these including cohousing groups, the ‘village’ movement, and the development of coproduction and coresearch (Buffel and Phillipson 2024). Taken together, they suggest alternative ways of ‘thinking about’ and ‘practising’ ageing: areas of innovation that can feed back into a different type of urban ageing, one which involves a *re-imaging of ageing in place as a broad social and community endeavour rather than an individual route into old age.*

The different types of work beginning to emerge also reflect a response to the diverse inequalities and pressures in urban environments. Dawson (2017, p. 6) highlights the emergence of what she terms ‘extreme cities’, describing them as urban areas marked by stark economic inequality. She views this phenomenon as a defining feature of contemporary urban environments and a significant threat to the sustainability of urban living. More generally, population ageing has coincided with the decline in essential social infrastructure within cities—libraries, community centres, affordable housing—negatively impacting quality of life and social relationships in general (Yarker 2022). This decline has underlined the need for collaborative forms of organisation that can foster social integration and address the fragmentation of community life (Hertz 2020).

Given the above context, the examples reviewed below represent the beginning of a debate about how to develop new ways of supporting ageing in place. The illustrations provided reflect the main types of collaborative and coproduced organisations which have emerged over the past two decades. The discussion draws on research which has assessed the strengths and limitations of the different community-based models which have emerged. Following the assessment of each model, the paper considers the next steps which need to be taken in supporting ageing in place as a collaborative venture.

## Building collaborative organisations for later life

This section examines four initiatives highlighting diverse approaches to strengthening community support to assist ageing in place, all of which involve different degrees of engagement from older people themselves: the *Village model*, *Naturally Occurring Retirement Communities*, *Cohousing, and Compassionate Communities*.

### The village model

The village model has been defined as ‘Self-governing, grassroots community-based organisations, developed with the sole purpose of enabling people to remain in their communities as they age’ (Scharlach and Lehning 2013, p. 119). Originating in Boston’s Beacon Hill neighbourhood in 2001, over 250 villages had been established in the USA by 2021, with an additional 100 in development (Village to Village Network 2022). Villages connect older residents living across a neighbourhood, drawing on the benefits of collective organisation to arrange support, services, and activities for themselves as a group. Membership fees (around $600 for individuals and $900 for households in 2021) enable services like transportation, companionship, home maintenance, technology assistance, and health care advocacy (Greenfield 2013). Villages also foster social engagement through events, activities, educational classes, and civic volunteering opportunities (Graham et al. 2014). The majority of villages are resident-led, initiated by, and having ongoing input from older people.

What are the benefits of the village model for supporting ageing in place? A significant dimension concerns the value of bringing people together in a locality, drawing on their experiences and resources to improve the lives of both the individuals concerned and the community as a whole. Scharlach et al. (2014) observed that over one-third of the villages (in their survey) were actively working to make their communities more age-friendly. This included improvements to both the physical and social environments, benefiting village members and the wider community. Additionally, bringing people together can yield practical advantages, such as enhancing the purchasing power of village members. Indeed, the authors found that nearly 40% of villages had negotiated discounts with external service providers for their members. This increased purchasing power could help secure higher quality services at lower costs and potentially improve the quality of goods and services available to other older residents in the area.

But questions have also been raised about the limitations of the village model in terms of the underrepresentation of minority groups and the extent to which membership fees restrict access to more affluent groups (Goff and Doran 2024). Research by Goff et al. (2020), which attempted to implement the village approach in two low-income communities in Manchester (UK), identified funding issues as a significant obstacle in developing the model, along with difficulties in recruiting volunteers for certain projects. Graham et al. (2014) found that while self-reported impacts of the village model are generally positive, especially with regards to social engagement and service access, its effectiveness in meeting the needs of the most vulnerable seniors remains uncertain. Nationally, villages predominantly attract members who are White, economically secure, and have relatively low levels of disability—i.e. those with the lowest risk of institutionalisation.

Despite these concerns, the village model has considerable potential in supporting people to age in place, harnessing the collective power of people living within a community. There are particular organisational benefits such as bulk purchasing of essential goods, health and social care advocacy, coordinating assistance with transportation, technology support, and organising access to vetted service providers. Nonetheless, the reliance on volunteer availability and engagement is a potential limitation of this model, as is the limited success in recruiting underrepresented groups (Davitt et al. 2017).

### Naturally occurring retirement communities

Another notable ageing in place model developed in the USA falls under the banner of naturally occurring retirement communities (NORCs). A NORC is a neighbourhood or building complex that was not originally designed for older adults but has gradually come to comprise a large proportion of people aged 60 or more. The NORC movement originated in the 1980 s in New York, where the dense living conditions in high-rise apartment buildings led to the emergence of early NORCs; this model subsequently expanded across New York City and State through the 1990 s and 2000 s (Jiaxuen et al. 2022). Building on this success, The Jewish Federation of North America initiated a campaign to expand NORC programmes nationwide, which now span rural, suburban, and urban neighbourhoods in the USA (Greenfield 2013). An additional feature of NORCs has been the development of what has been termed supportive service programmes (SSP), these integrating health and community services within schemes to offer programmes and activities which can strengthen ageing in place. As a result, and in contrast to villages, NORCs are often closely enmeshed with health and social services, with a roster of paid staff.

NORCs represent an important development in responding to the needs of what may be ‘unplanned’ communities of older adults—in neighbourhoods, tower blocks, or housing estates. Research has indicated a number of benefits associated with this type of approach, including reducing the likelihood of admission to residential or nursing home care (Elbert and Neufeld 2010); lowering the number of visits to accident and emergency centres, hospital visits, and the incidence of falls (NIA and NORC Innovation Center 2022); and strengthening the connections which older adults have within their communities and the wider social networks to which they might be linked (Jiaxuan 2022).

But the challenges facing NORCs also highlight some of the complexities of supporting ageing in place. The NORC model tends to prioritise leadership roles for health and social service professionals, resulting in often limited involvement of older people in key positions within NORCs (Parniak et al. 2022). Funding cuts have also been a constant challenge for this type of model (Greenfield 2013). Vladeck and Altman (2015) emphasise the necessity for a public vision which might facilitate the large-scale adoption of the NORC approach. However, they express doubt about whether the financial investment to support such expansion will be forthcoming. Finally, as with villages, questions about the inclusivity of NORCs have been raised. Davitt et al. (2017), in a survey of NORCs in New York combined with a national sample, found that programmes struggled with recruiting frailer or more isolated older adults. Despite being more ethnically diverse than villages, NORCs also encounter challenges in recruiting minority groups due to staffing issues and difficulties in providing translation services for older adults with limited English proficiency.

Both villages and NORCs offer different approaches to the challenge of supporting people who wish to age in place within their community. In the context of the USA (but applicable to many other countries), Mahmood et al. (2022, p. 72) suggest that: ‘Villages and NORC-SSP models add a critical piece missing in fragmented public health care systems by incorporating older adults’ access to health care services and supports…These two models also demonstrate how partnerships between local government, regional health authorities, and third sector organisations could help provide coordinated services and support to older residents’.

The next section discusses another example of collective action to support ageing in place, i.e. that of cohousing—intentional communities developed and governed by residents through collective and consensual-based decision-making.

### Cohousing

While villages and NORCs illustrate attempts to organise services in existing neighbourhoods, the cohousing model purposely creates new communities with shared services to meet the needs of families of all ages, including older adults. Hammond (2018) points to a growing cohort of older people seeking to develop cohousing as a way of responding to the opportunities and challenges of ageing. Arrigoitia and West (2021, p. 1673–1696) note that with its emphasis on mutual aid among residents: ‘cohousing has long been mooted as an alternative to the rather limited later life options of ageing in place in one’s familiar home, sheltered accommodation, extra-care, or residential and nursing home care’. As such, it aims to widen later life housing options beyond the dichotomy of ‘independent’ community living and ‘institutional’ care (Stoisser et al. 2025).

Cohousing first emerged in Denmark in the 1970 s and 1980 s, spreading out to various European countries and North America, albeit with faster take-up in some countries than others (Pedersen 2015). Cohousing communities are usually resident-led and managed, with the explicit aim of generating social bonds between residents. Hammond (2018) suggests that the increasing interest in this type of development can be linked to the transitions of aspirational baby boomers into older age, who seek an alternative to living alone while rejecting conventional forms of housing. He argues that a major advantage of cohousing for older adults is the opportunity to be open about their ageing experiences, thereby facilitating mutual support in response to changing physical capabilities and emotional needs as they age. Arrigoitia and West (2021), in an ethnographic study of the UK’s first older women’s cohousing community (New Ground), observed that the residents take pride in their ability to acknowledge and address the challenges of ageing, supporting each other through this process. They believe that a fulfilling old age is achieved through mutual commitment and community learning, rather than solely through conventional advice on diet and physical activity. Although these more conventional aspects are not completely absent from their discussions, they often rely on one another to share knowledge and ‘tips’ about illnesses and recovery.

‘Intentional communities’ such as cohousing appear to have many of the characteristics—sharing, mutual aid, collective support—deemed essential for providing more effective foundations to support ageing in place (Pedersen 2015). Yet the disadvantages of the model must also be noted. It remains, for a variety of reasons, a minority element across the spectrum of housing provision for later life (Stoisser et al. 2025). Difficulties in the development process are an important factor, with Hammond (2018) citing evidence for the UK that just one in 10 cohousing groups ever progress to the construction phase, often taking 10 or more years to complete but with many schemes failing to get off the ground. Lack of planning expertise, and difficulties procuring land are among the major challenges facing prospective groups. Such issues are especially pertinent for older people’s cohousing, where a prolonged development process might account for a significant portion of the individuals’ remaining years.

Such difficulties appear to be reinforced through ageist attitudes towards older people seeking new ways of living in later life. Arrigoitia and West (2021), in their account of *New Ground*, a UK cohousing scheme developed by a group of older women (which itself took 18 years from conception to people moving into the scheme) note that developers and housing associations struggled to listen to and collaborate creatively with older individuals, especially older women. Local authorities tended to view the scheme as a potential burden on public care finances rather than a model for enhanced cocare and healthier living for older adults. More generally, the time and resources to develop cohousing almost certainly make it an option restricted to people with high combinations of financial and social capital. In Denmark, where cohousing first developed, the evidence appears to be that it has evolved into: ‘enclaves for the relatively privileged’ (Larsen 2019, p. 34). This form of cohousing, primarily based on owner-occupation, tends to contribute to the commodification of housing and land, a fundamental cause of social and spatial inequalities in Denmark and elsewhere.

### Compassionate communities

Finally, another important initiative has been work coming under the heading of ‘*Compassionate Communities*’, these defined as: ‘A community of people who are passionate and committed to improving the experiences and well-being of individuals who are dealing with a serious health challenge, and those who are caregiving, dying, or grieving’ (D’er et al. 2022, p. 626). Members of a compassionate community actively support individuals impacted by these experiences by linking them to valuable resources, raising awareness about end-of-life matters, and creating supportive networks within the community. Vanderstichelen et al. (2022) observe that the compassionate communities approach has rapidly gained traction, with initiatives appearing across both the Global North and South. They highlight that while there is important literature on dying in place, the community’s role in achieving this outcome has not been fully explored.

Indeed, it is certainly the case that there has been limited consideration of the needs of individuals with a terminal illness or those experiencing bereavement in age-friendly and age in place activities. D’er et al. (2022, p. 24) conducted a systematic review on what they termed ‘civic engagement’ in supporting people facing serious illness and death, noting that these initiatives commonly involve engaging communities in linking individuals with palliative care needs with community members who can offer help. This approach differs from the typical service-centred model that focuses primarily on clinical interventions and treating illness. Instead, compassionate communities adopt a salutogenic approach, aiming to enhance overall well-being through health promotion. Consequently, these initiatives emphasise community-based social support for those dealing with illness, death, and loss. We would argue for the compassionate communities approach to be embedded in the other approaches discussed in this section, and to be disseminated more widely in age-friendly communities and networks.

## Ageing in place as a collaborative venture: Developing a new agenda

Ageing in place has emerged as a dominant theme in public policies directed towards older people, reflecting the shift from focussing on *‘care for’* to ‘*care by’* the community, with greater emphasis on the supportive role of family, friends, and neighbours (Means et al. 2008). Despite its prominence in research and policy, the implementation of ageing in place often lacks systematic planning or financial support. This raises questions about the types of organisations and relationships which need to be developed to ensure access to the resources required to support ageing in place (Buffel and Phillipson 2024).

To address these issues, it is essential to develop a new agenda for ageing in place. This agenda should move beyond the traditional view of ageing in place as an issue solely focussed on individuals or their families. Instead, it should encompass a broader perspective that examines how older adults maintain and develop connections within their communities. Key questions to consider include: *How effectively do these connections promote the mobilisation of resources needed to support ageing in place? To what extent are these networks socially inclusive? And how can older people influence broader economic and social processes that affect the quality of life in their communities?* Recognising that ageing in place cannot be achieved in isolation but requires reciprocal interaction and interdependence will be crucial in developing a new approach to ageing in place as a collaborative venture*.*

The examples discussed in this paper provide different ways of addressing these questions. Villages, NORCs, cohousing, and compassionate communities share a common focus on building relationships centred around elements of sharing, mutual aid, and collective support. Such initiatives acknowledge that ageing in place is difficult to achieve ‘alone’; instead, it necessitates reciprocal interaction, assistance, and *interdependence*. This insight is especially significant given that single households—across all age groups—represent the fastest-growing demographic across all types of communities in different countries. So, the issue of how best to ‘organise’ for ageing in place is an important one. It is of particular significance given the range of challenges affecting urban communities outlined in this paper. Indeed, building collective organisations for later life are especially important to resist pressures associated with the privatisation of public space, the erosion of social infrastructure, and what Kelley et al. (2018, p. 58) view as ‘the potential [of older adults] to be erased or rendered invisible, in their own neighbourhoods’.

Despite the merits of the examples discussed in helping to increase the visibility of older adults in urban environments, their limitations are also significant: first, in respect of their ‘exclusivity’ by potentially omitting particular groups, including minorities, people with long-term conditions, and those in precarious financial circumstances. Second, challenges relating to scale and limited influence over the larger-scale pressures affecting the capacity of these organisations to deliver services. Third, ensuring the financial sustainability of organisations typically dependent upon grants and voluntary support. Additional innovations and strategies are therefore necessary both to influence the broader *environment* in which organisations supporting ageing in place are operating, and to ensure that interventions are *inclusive* of the *diversity* of older adults. To this end, three key strategies will be explored: *first, developing an anti-ageist approach to urban planning; second, developing coproduction within communities; third, developing new forms of collaborative organisation*.

### Developing an anti-ageist approach to urban planning

To ensure that organisations supporting ageing in place are genuinely inclusive, it is essential to examine how urban planning practices can either promote or impede this inclusivity. One significant issue is the prevalence of ageism within urban development strategies. Often, efforts to stimulate economic growth in cities implicitly or explicitly prioritise certain groups, such as the ‘creative classes’, ‘students’, ‘young professionals’, and ‘wealthy elites’, while neglecting older adults (Kern 2022). Initiatives to brand cities as ‘age-friendly’ may thus be at odds with the core groups that mayors and developers want to attract, a point echoed by Hammond et al. (2024) who term this as a form of ‘spatial ageism’. Their analysis highlights how ageist biases among architects, planners, and developers can result in exclusionary and age-segregated urban environments that prioritise ‘*productivity-focussed urban planning*’. By taking a view that older people are not productive economic actors, urban plans tend to favour the needs and aspirations of younger adults, emphasising job creation and graduate retention for economic growth while neglecting older people who receive little to no benefits from wider urban regeneration efforts.

Kim and Cho (2018, p. 99), reflecting on their experience of the impact of urban regeneration on older people in a neighbourhood in Seoul, South Korea, suggest that an age-friendly community will require: ‘a paradigm shift in the public discourse on ageing and public space’. They conclude:

‘As ageing is a “normal” life stage, a city is also a place of natural ageing and older urban dwellers. The advocacy for the active engagement of older people reflects the reality that the consideration of older urban dwellers has so far been absent in urban policies, which are predominantly centred on the working-age populace…Older residents’ desire to age-in-place can be seen as a positive factor to imagine urban development—one which is more sensitive towards the needs of older people and places more emphasis on progressive transformation compared to large-scale urban development driven by property development’.

Sharing urban planning strategies with different groups within the older population will be essential, with awareness of contrasting issues faced by different ethnic groups, those with particular physical and mental health needs, and those living in areas with higher levels of economic and social deprivation (Kavanagh and Lewis 2024). At the same time, age-aware urban planning should not only focus on changes for current cohorts of older residents but also work towards longer-term neighbourhood change that can benefit successive cohorts of older residents. Therefore, there is an urgent need to reconnect urban planning to strategies that support resident-led work around the development of *life-time* neighbourhoods and communities, as well as interventions that expand the range of social infrastructure supporting ageing in place (Buffel and Phillipson 2024). This will involve public and private sectors and voluntary and community organisations working together so that residents of all ages can articulate their needs and concerns and identify priorities for action and change within their neighbourhoods and the urban environment in which it is embedded.

### Developing coproduction within communities

Second, engaging older people as key participants in urban development requires systematic attention to what is termed ‘coproduction’ as a mechanism for affecting change and involving older adults more centrally in the decisions that shape their communities (Buffel 2019). This approach aims to put principles of ‘empowerment’ and ‘participation’ into practice, working ‘with’ communities and offering residents greater control over their environment. It builds on a partnership between older people, their families, communities, statutory, and non-statutory organisations who work together to jointly develop research and a shared understanding, as well as to design, develop, and deliver opportunities, projects, and solutions promoting social and political change (James and Buffel 2023; Urbaniak and Wanka 2023). In this sense, coproduction methods are at the heart of developing age-friendly policies and initiatives: among other stakeholders, older people are recognised as key actors in developing research and action to assist in planning or modifying the environments in which they live. Smetcoren et al. (2018) explored these issues in their work in Brussels developing what they termed ‘active caring communities’. They reported how the professional workers interviewed in their research:

‘…highlighted the need to promote a better understanding of how neighbourhood support networks can play a role in supporting older people to age in place and supporting what neighbours at present already contribute to the care needs of frail older people. According to some participants, when creating an age-friendly social environment, the focus *should not* be on the development of new social networks, but rather on making existing networks visible and supporting and valorising them’ (Smetcoren et al. 2018, p. 109).

Recognising older people as actors in the social environment is essential to creating age-friendly communities (Doran and Buffel 2018). The fundamentally subjective nature of communities, and the importance of negotiating one’s local environment, make empowerment and recognition of older resident’s paramount to achieving age-friendliness. This implies investment in working with older residents as key partners in designing policies, especially for vulnerable and isolated groups within the community (van Hoof et al. 2021). Methods of coproduction and coresearch have been proven useful in engaging such groups and have gained ground in the development of health and welfare services. Information and communication technologies may also support the involvement of older residents in navigating and designing their environment (Marston et al. 2023).

The case for coproduction methods with older people in developing age-friendly cities and communities may be summarised as threefold (Buffel 2019): firstly, it represents a viable method to working with older residents and mobilising their expertise, skills, and knowledge to stimulate creative reform ideas and initiatives around the age-friendliness in their neighbourhood; secondly, it makes older people themselves central to the creation and development of policies and age-friendly initiatives; finally, coproduction offers a range of benefits to the different stakeholders involved, providing a forum for rich and meaningful social engagement and mutual learning and exchange.

At the same time, it is important to note that coproduction is increasingly challenged by the inequalities within the older population and power differentials within and between groups. It is hard to recruit older people to engage in coproduction, particularly when working with marginalised groups, as illustrated in a study which aimed to implement the village model in deprived communities in Manchester (Goff et al. 2020; Goff and Doran 2024). It is often those already engaged in activity in their communities that take part in coproduction. Therefore, such work can run the risk of creating a further divide between an already more ‘privileged’ group of older people and their more disadvantaged peers. It is vital that power differences created through the coproduced work, as well as ethical challenges and cross-cutting issues of gender, class, race, and sexual orientation, are made explicit and reflected on across the process as it unfolds (Buffel 2019; Ubaniak 2023). Developing a coproduction dimension to dementia-friendly communities is also a priority, with the need to involve people with dementia as chairs of meeting, contributing to steering groups, and supporting work on the design of their outdoor environment.

### Developing new forms of collaborative organisation

Finally, it will be important to continue to experiment with new forms of collaborative organisation. All the examples discussed in this chapter (with the exception of compassionate communities) have existed since the 1980 s and 1990 s, and fresh approaches are now needed given the changes described in this paper. Older people face a more challenging environment given the long-term impact of COVID-19, continued cuts to core health and social care services, and greater diversity among older adults in respect of economic, health, and social conditions. And, as has been argued in this paper, the urban context itself presents major challenges for supporting ageing in place, with contrasting waves of gentrification and disinvestment de-stabilising many communities (Kern 2022).

But all these developments are essentially arguments *for* strengthening collaborative organisations to underpin ageing in place. Ageing in place may be a preference for most older people—though many also migrate to another place or need a more supportive place later in life—but for those who stay, there may be a considerable gap between their own needs and the support available within the community. Collaborative organisations may themselves need to change in response: for example, by fostering new partnerships with public, private and voluntary health and social care organisations; developing intergenerational coalitions to press the case of improvements to public services; and by expanding the range of housing options available for those on low incomes and/or with significant physical disabilities. These issues point to the range of issues likely to be faced in the next phase of policies to promote ageing in place, where it is crucial that older people should take a leading role in shaping their development at local, regional, and national levels.

## Conclusion

This paper has reviewed evidence concerning the strengths and limitations of collaborative activity to support the policy of ageing in place. The discussion has also highlighted structural inequalities affecting urban environments, and the negative impact of these on the physical and social infrastructure supporting diverse older populations. But, as indicated at different points, this situation has arisen through what remains the continued ‘invisibility’ of older people in place-making policies, with priority going to groups perceived as having greater economic and social value. Challenging this approach will require *making ageing in place a normal part of what happens in a city, to be planned for alongside the full range of cultural, economic, and social activities*. In this way, ageing populations can play their part in re-purposing cities in the twenty-first century, drawing on the benefits of sharing and mutual aid which effective support for ageing in place must entail. Far from being a marginal group in the daily life of cities, our argument is for older people to have an equal share of the decisions and processes that impact the quality and organisation of daily life.

## Data Availability

No datasets were generated or analysed during the current study.

## References

[CR1] Andrews GJ (2024) Geographical gerontology: knowing place in five ways. In: Cutchin M, Rowles GD (eds) Handbook on ageing and place. Edward Elgar, Massachusetts

[CR2] Arrigoitia AM, West K (2021) Independence, commitment, learning and love: the case of the United Kingdom’s first older women’s co-housing community. Ageing Soc 41(7):1673–1696. 10.1017/S0144686X19001673

[CR3] Buffel T (2019) Older co-researchers exploring age-friendly communities. An ‘insider’ perspective on the benefits and challenges of peer-research. Gerontologist 59(3):538–548. 10.1093/geront/gnx216 29401222 10.1093/geront/gnx216

[CR4] Buffel T, Phillipson C (2019) Ageing in a gentrifying neighbourhood: experiences of community change in later life. Sociology 53(6):987–1004. 10.1177/0038038519836848

[CR5] Buffel T, Phillipson P (2024) Ageing in place in urban environments: Critical perspectives. Routledge, London

[CR6] Buffel T, Doran P, Yarker S (2024) Reimagining age-friendly communities: urban ageing and spatial justice. Policy Press, Bristol

[CR7] D’er L, Quintiens B, Van den Block L, Dury S, Deliens L, Chambaere K, Smets T, Cohen J (2022) Civic engagement in serious illness death, and loss: a systematic mixed-methods review. Palliat Med 36:625–651. 10.1177/02692163221077850 35287517 10.1177/02692163221077850 PMC11910752

[CR8] Davitt JE, Greenfield E, Lehning A, Scharlach A (2017) Challenges to engaging diverse participants in community-based ageing in place initiatives. J Community Pract 25:325–343. 10.1080/10705422.2017.1354346

[CR9] Dawson A (2017) Extreme cities: the peril and promise of urban life in the age of climate change. Verso Books, London

[CR10] Doran P, Buffel T (2018) Translating research into action: involving older people in co-producing knowledge about age-friendly neighbourhoods. Work Older People 22(1):39–47. 10.1108/WWOP-11-2017-0033

[CR11] Elbert KB, Neufeld PS (2010) Indicators of a successful naturally occurring retirement community: a case study. J Hous Elderly 24(3–4):322–334. 10.1080/02763893.2010.522440

[CR12] Florida R (2017) The new urban crisis: gentrification, housing bubbles, growing inequality, and what we can do about it. Oneworld Books, London

[CR13] Goff M, Doran P (2024) Co-producing age-friendly community interventions: The Village model. In: Buffel T, Doran P, Yarker S (eds) Reimagining age-friendly communities: urban ageing and spatial justice. Policy Press, Bristol, pp 118–134

[CR14] Goff M, Doran P, Phillipson C, D’Andreta D (2020) Community interventions to promote ‘ageing in place’: developing the ‘Village’ model in Manchester. Manchester Institute for Collaborative Research into Ageing, Manchester

[CR500] Golant, S. (2015) Aging in the Right Place. Health Professions Press. Brookes Publishing Company, Baltimore.

[CR15] Golant SM (2024) Dwellings occupied by mobility-limited older people emerge as strong control centres and more age-friendly places. J Aging Stud 70:101245. 10.1016/j.jaging.2024.101245 39218493 10.1016/j.jaging.2024.101245

[CR16] Goldin I, Lee-Devlin T (2023) Age of the city: why our future will be won or lost together. Bloomsbury, London

[CR17] Graham S (2016) Vertical: the city from satellites to bunkers. Verso Books, London

[CR18] Graham C, Scharlach A, Wolf JP (2014) The impact of the ‘Village’ model on health, well-being, service access, and social engagement of older adults. Health Educ Behav 41(1):91–97. 10.1177/1090198114532 10.1177/1090198114532290 24799128

[CR19] Greenfield EA (2013) The longevity of community aging initiatives: a framework for describing NORC programs’ sustainability goals and strategies. J Hous Elderly 27(1–2):120–145. 10.1080/02763893.2012.754818

[CR20] Greenfield E, Buffel T (2022) Age-friendly cities and communities: research to strengthen policy and practice. J Aging Soc Policy 34(2):161–174. 10.1080/08959420.2022.2049573 35311484 10.1080/08959420.2022.2049573

[CR503] Hammond M (2018) Spatial Agency: Creating New Opportunities for Sharing and Collaboration in Older People's Co-Housing. Urban Science 2(3):64. 10.3390/urbansci2030064

[CR21] Hammond M, Crompton E, White S (2024) Redesigning the age-friendly city: the role of architecture in addressing spatial ageism. In: Buffel T, Doran P, Yarker S (eds) Reimagining age-friendly communities: Urban ageing and spatial justice. Policy Press, Bristol, pp 135–155

[CR22] Handler S (2014) The alternative age-friendly handbook. University of Manchester Library, Manchester

[CR23] Hertz N (2020) The lonely century: coming together in world that’s pulling apart. Sceptre, London

[CR24] Hillcoat-Nallétamby S, Ogg J (2014) Moving beyond ‘ageing in place’: Older people’s dislikes about their home and neighbourhood environment as a motive for wishing to move. Ageing Soc 34(10):1771–1796. 10.1017/S0144686X13000482

[CR25] Horner B, Boldy D (2008) The benefit and burden of ‘ageing-in-place’ in an AgedCare community. Aust Health Rev 32(2):356–365. 10.1071/AH080356 18447827 10.1071/ah080356

[CR26] Jiaxuan E, Xia B, Susilawati C, Chen Q, Wang X (2022) An overview of naturally occurring retirement communities (NORCs) for ageing in place. Buildings 12(5):519. 10.3390/buildings12050519

[CR27] Kavanagh N, Lewis C (2024) Developing age-friendly communities in areas of urban regeneration. In: Buffel T, Doran P, Yarker S (eds) Reimagining age-friendly communities: Urban ageing and spatial justice. Policy Press, Bristol, pp 100–117

[CR28] Kelley J, Dannefer D, Masarweh L (2018) Addressing erasure, microfication and social change. In: Buffel T, Handler S, Phillipson C (eds) Age-friendly cities and communities: a global perspective. Policy Press, Bristol, pp 51–72

[CR29] Kern L (2022) Gentrification is inevitable. And other lies. Verso Books, London

[CR30] Kim J, Cho M (2018) Fostering government-Citizen collaboration and inter-generational cooperation: the alternative neighbourhood regeneration project in Jangsu, Seoul. In: Chong KH, Mihye C (eds) Creative ageing cities: place design with older People in Asian cities. Routledge, London, pp 95–117

[CR31] Larsen HG (2019) Denmark: Anti-urbanism and segregation. In: Hagbert P, Larsen HG, Thörn H, Wasshede C (eds) Contemporary co-housing in Europe: towards sustainable cities. Routledge, London, pp 23–37

[CR32] Mahmood A, Seetharaman K, Jenkins HJ, Chaudhury H (2022) Contextualizing innovative models and services within the age-friendly communities framework. Gerontologist 62(1):66–74. 10.1093/geront/gnab115 34355769 10.1093/geront/gnab115

[CR33] Marston HR, van Hoof J, Yon Y (2023) Digitalising the built environment for all generations: a new paradigm for equity and inclusive age-friendly cities and communities. Indoor Built Environ. 10.1177/1420326X231176621

[CR34] Means R, Richards S, Smith R (2008) Community care: policy and practice. Palgrave Macmillan, Basingstoke

[CR35] National Institute on Ageing and NORC Innovation Centre (2022) It’s time to unleash the power of Naturally Occurring Retirement Communities in Canada. ON: National Institute on Ageing, Toronto Metropolitan University and NORC Innovation Centre, University Health Network, Toronto

[CR36] Neamtan N (2005) The social economy: Finding a way between the market and the state. Policy Options. https://policyoptions.irpp.org/magazines/sustainable-development/the-social-economy-finding-a-way-between-the-market-and-the-state/. Accessed 1 July 2023

[CR37] OECD (Organisation of Economic Cooperation and Development) (2021) Demographic trends. In Health at a glance 2021: OECD indicators. OECD Publishing, Paris

[CR38] Oswald F, Wahl H-W, Wanka A, Chaudhury H (2024) Theorizing place and aging: enduring and novel issues in environmental gerontology. In: Cutchin M, Rowles GD (eds) Handbook on ageing and place. Edward Elgar, Massachusetts

[CR39] Parniak S, DePaul VG, Frymire C, DePaul S, Donnelly C (2022) Naturally occurring communities: scoping review. JMIR Aging 5(2):1–14. 10.2196/34577 10.2196/34577 PMC9052023 35436204

[CR40] Pedersen M (2015) Senior co-housing communities in Denmark. J Housing Elderly 29(1–2):126–145. 10.1080/02763893.2015.989770

[CR41] Rowles GD, Cutchin MC (2024) Envisioning a future: toward an agenda for the study of aging and place. In: Cutchin M, Rowles GD (eds) Handbook on ageing and place. Edward Elgar, Massachusetts

[CR42] Russel E (2024) Rural places and aging: between aging in place and stuck in place. In: Cutchin M, Rowles GD (eds) Handbook on ageing and place. Edward Elgar, Massachusetts

[CR43] Savage M (2021) The return of inequality: Social change and the weight of the past. Harvard University Press, Cambridge

[CR44] Scharlach AE, Lehning AJ (2013) Ageing-friendly communities and social inclusion in the United States of America. Ageing Soc 33(1):110–136. 10.1017/S0144686X12000578

[CR45] Scharlach AE, Davitt JK, Lehning AJ, Greenfield EA, Graham CL (2014) Does the Village model help to foster age-friendly communities. J Aging Soc Policy 26(1–2):181–196. 10.1080/08959420.2014.854664 24224776 10.1080/08959420.2014.854664

[CR46] Smetcoren A-S, De Donder L, Duppen D, De Witte N, Vanmechelen O, Verté D (2018) Towards an ‘active caring community’ in Brussels. In: Buffel T, Handler S, Phillipson C (eds) Age-Friendly cities and communities: a global perspective. Policy Press, Bristol, pp 97–118

[CR47] Smetcoren A-S (2015) I’m not leaving!?’ Critical perspectives on ageing in place. Vrije Universiteit Brussel, Brussels

[CR48] Stein S (2019) Capital city: gentrification and the real estate state. Verso Books, London

[CR49] Stoisser L, Buffel T, Petermans A, Smetcoren A-S (2025) Rethinking community-based housing for older adults: a research agenda for spatial justice. Int J Hous Policy. 10.1080/19491247.2025.2515641

[CR50] United Nations (2019) World urbanization prospects. The 2018 revision. United Nations, New York

[CR51] Urbaniak A (2023) Role of reflexivity in challenging participation inequality in participatory approaches with older adults. In: Urbaniak A, Wanka A (eds) Routledge International handbook of participatory approaches in ageing research. Routledge, London, pp 17–30

[CR52] Urbaniak A, Wanka A (2023) Routledge international handbook of participatory approaches in ageing research. Routledge, London

[CR53] van Hoof J, Marston HR, Kazak JK, Buffel T (2021) Ten questions concerning age-friendly cities and communities and the built environment. Build Environ 199:107922. 10.1016/j.buildenv.2021.107922

[CR54] Vanderstichelen S, Dury S, De Gieter S, Van Droogenbroeck F, De Moortel D, Van Hove L, Rodeyns J et al (2022) Researching compassionate communities from an interdisciplinary perspective: the case of the compassionate community centre of expertise. Gerontologist 62(10):1392–1401. 10.1093/geront/gnac034 35263765 10.1093/geront/gnac034

[CR55] Village to Village Network (2022) Village to village network helps communities establish and operate thriving villages. The village-to-village network. www.vtvnetwork.org/content.aspx?page_id=22&club_id=691012&module_id=248579. Accessed 3 December 2023.

[CR56] Vladeck F, Altman A (2015) The future of the NORC-supportive service program model. Public Policy Aging Rep 25(1):20–22. 10.1093/ppar/pru050

[CR57] Wahl HW, Oswald F (2010) Environmental perspectives on ageing. In: Danner D, Phillipson C (eds) The SAGE handbook of social gerontology. SAGE, London, pp 111–124

[CR58] Wilson B (2020) Metropolis: a history of humankind’s greatest invention. Jonathan Cape, London

[CR501] World Health Organization (2007) Global Age Friendly Cities: A Guide. WHO, Geneva

[CR59] World Health Organization (2018) The global network for age-friendly cities and communities. WHO, Geneva

[CR60] World Health Organization (2020) Decade of healthy ageing: Baseline report. www.who.int/publications/i/item/9789240017900.

[CR61] Wright EO (2010) Envisioning real utopias. Verso Books, London

[CR62] Yarker S (2022) Creating spaces for an ageing society: the role of critical social infrastructure. Emerald Publishing Limited, Bingley

[CR63] Yarker S, Doran P, Buffel T (2023) Theorizing ‘place’ in aging in place: the need for territorial and relational perspectives. Gerontologist. 10.1093/geront/gnad002 10.1093/geront/gnad002 PMC10809216 36655690

